# Cost-Effective Laser Powder Bed Fusion of Ti-6Al-4V Grade 5: The Effect of Expanding Powder Size Distribution on Mechanical Performance

**DOI:** 10.3390/ma18010006

**Published:** 2024-12-24

**Authors:** Min Soo Kim, Ohseop Kim, Youngwon Song, Chanyoung Ko, Jonggun Kim, Mahdi Habibnejad-korayem, Jeong Ho Kim

**Affiliations:** 1 Department of Aerospace Engineering, Inha University, Incheon 22212, Republic of Korea; kms.ascent@inha.edu (M.S.K.); seop8936@inha.edu (O.K.); 2 Institute for Aerospace Industry-Academia Collaboration (IAIAC), Incheon 22212, Republic of Korea; ywsong@iaiac.or.kr (Y.S.); cyko0908@iaiac.or.kr (C.K.); 3 Korea Aerospace Industries (KAI), Sacheon 52529, Republic of Korea; jgunkim@koreaaero.com; 4 AP&C, a—Additive Company, GE Aerospace, Saint-Eustache, QC J7R 0L5, Canada; mahdi.habibnejad@geaerospace.com

**Keywords:** laser powder bed fusion (LPBF), Ti-6Al-4V grade 5, powder size distribution (PSD), mechanical properties, cost reduction

## Abstract

This study aims to assess the feasibility of expanding the powder size distribution (PSD) of Ti-6Al-4V grade 5 powder for LPBF to achieve cost reduction. Parameter optimization to minimize the degradation of mechanical properties due to the expanded particle size distribution was conducted. Mechanical tests for specimens built using optimized parameters revealed minor reductions in strength: 3.9% in tensile yield strength, 1.1% in compressive strength, 5.5% in shear strength, and 4.5% in bearing yield strength—all of which complied with MMPDS standards. Statistical analysis, using the Anderson–Darling test, demonstrated stable mechanical performance and minimal variation between the original and expanded PSDs. These results highlight the potential of an expanded PSD to achieve cost reductions while maintaining compliance with industry standards, offering a practical solution for LPBF applications in cost-sensitive and high-performance industries.

## 1. Introduction

Additive manufacturing (AM) technologies offer innovative solutions across industries by enabling the precise and rapid production of complex structures. Among these, powder bed fusion (PBF) has gained prominence in sectors like aerospace, automotive, and medical devices for its precision and efficiency [1,2,3,4,5,6,7]. In particular, laser-powder bed fusion (L-PBF), a specific PBF method, allows the creation of high-accuracy parts with precision levels as fine as 10 µm, enabling complex-shaped parts by selectively melting powder materials layer by layer using a laser [8,9,10,11].

Ti-6Al-4V grade 5, a titanium alloy known for its high strength, corrosion resistance, and excellent performance in high-temperature environments, is widely used in aerospace, prosthetics, and other high-value industries [12,13,14]. Despite its advantageous properties, the inherently high cost of titanium alloy powders—combined with the challenging machining requirements and the need for specialized equipment—significantly increases the price of Ti-6Al-4V parts, limiting its broader application [15,16,17,18,19].

Conventionally, this material has a tightly controlled powder size distribution (PSD) (15–53 μm), which contributes to material waste and higher logistics costs [20,21,22,23,24]. By expanding the PSD to 15–75 μm, sometimes termed the wide PSD in the literature, the production process can be simplified and cost savings can be achieved while maintaining process stability. In this context, the connection between material cost reduction and mechanical performance becomes critical for assessing the feasibility of PSD expansion.

Previous research highlights the influence of PSD on manufacturing processes and product quality. Soltani-Tehrani et al. [25] found that parts fabricated using finer powders exhibit more consistent quality, while Rauniyar et al. [26] demonstrated that a narrower PSD improves melt pool stability and surface finish. Additionally, finer powders and optimized laser parameters can significantly influence surface macrotexture, affecting key tribological properties such as friction coefficient and wettability [27]. However, Meier et al. [28] noted that excessively fine powders may cause cohesion issues, affecting surface quality, and Chu et al.’s work [29] demonstrated that coarser powders may enhance relative density. These studies indicate that PSD plays a crucial role in product quality, but its potential for cost reduction has not been fully explored.

Ludwig et al. [30] demonstrated that expanding PSD can significantly contribute to cost reduction. Their studies revealed the potential for savings by applying the coarser PSD (45–106 µm) of Ti-6Al-4V, typically used in EB-PBF, to the L-PBF process. Ludwig’s findings emphasize that manufacturing cost savings are achievable with a broader PSD if mechanical properties, such as yield strength, tensile strength, and elongation, can be preserved, a critical insight for advanced industries where the high cost of metal powders presents a substantial barrier. According to evaluations by the powder manufacturer Advanced Powder & Coating (AP&C, Saint-Eustache, QC, Canada), expanding the PSD from 15–53 μm to 15–75 μm may lead to reduce powder costs by roughly 20%.

However, while prior research has demonstrated the general benefits and challenges of PSD expansion, there is a lack of comprehensive studies examining the direct impact of expanded PSD on the mechanical properties and process stability of Ti-6Al-4V grade 5. It remains unclear how PSD expansion affects key performance factors, such as tensile strength, compression strength, and shear resistance, when applied to this widely used alloy.

To address these gaps, the present study focuses on the systematic evaluation of these relationships, ensuring that both performance and cost considerations are tightly interwoven throughout the research. This study experimentally expands the PSD of Ti-6Al-4V grade 5 from 15–53 μm to 15–75 μm and examines its impact on process efficiency and cost reduction. To achieve this, new energy density optimization parameters were developed, and comprehensive mechanical tests—including tension, compression, shear, and bearing evaluations—were conducted to assess the mechanical performance of the expanded PSD. The connection between these performance indicators and material cost savings was carefully analyzed to establish a consistent narrative. Additionally, statistical analysis was performed to confirm the reliability and stability of the results, as demonstrated by achieving compliance with MMPDS standards. These findings aim to provide valuable insights into improving cost efficiency and material utilization in L-PBF processes. Ultimately, the goal of this study is to explore the feasibility of cost reduction and process efficiency improvements in L-PBF by expanding the PSD of Ti-6Al-4V grade 5.

## 2. Procedure

### 2.1. Equipment

The equipment used in this study is the M2 Dual-Laser system by Colibrium Additive (West Chester, OH, USA). The system features two lasers, each with a maximum output of 400 W, and is optimized for processing Ti-6Al-4V grade 5 material. The M2 system allows for high-density metal additive manufacturing through its high-powered lasers and offers adjustable laser parameters. In this study, the laser parameters were adjusted to achieve optimal results for melting powder particles with sizes up to 75 µm. Table 1 provides the specifications of the M2 equipment used in this study.

### 2.2. Material

The Ti-6Al-4V grade 5 material for this study was provided by AP&C. AP&C is a certified supplier for aerospace component quality, holding both ISO 9100 [31] and AS9100D [32] certifications, ensuring high-quality powder. The powder used in this study was produced through the advanced plasma atomization (APA™) process, which maintains optimal powder morphology and physical properties that ensure high consistency for additive manufacturing applications compared to other powder manufacturing processes. Table 2 summarizes the material specifications and certification details for the Ti-6Al-4V grade 5 powder used in this study.

This study focuses on two PSD ranges of the same powder: the original PSD (15–53 μm) and an expanded PSD (15–75 μm). While L-PBF processes typically favor narrower PSDs like 15–53 μm for consistent melting and bonding, achieving this range requires significant refinement steps. These processes reduce the yield of usable powder, increase waste, and add costs associated with labor, equipment, and recycling.

The expanded PSD (15–75 μm) allows the direct use of particles within the 53–75 μm range that would traditionally be discarded. This adjustment aims to increase the usable powder production yield and reduce powder waste and associated costs. Furthermore, this strategy has a positive impact on reducing the carbon footprint of additive manufacturing. By incorporating larger particles, the expanded PSD improves the frequency of sieving, reduces equipment wear, and lowers maintenance costs, ultimately decreasing the total cost of ownership (TCO) of powder production.

**Figure 1:**
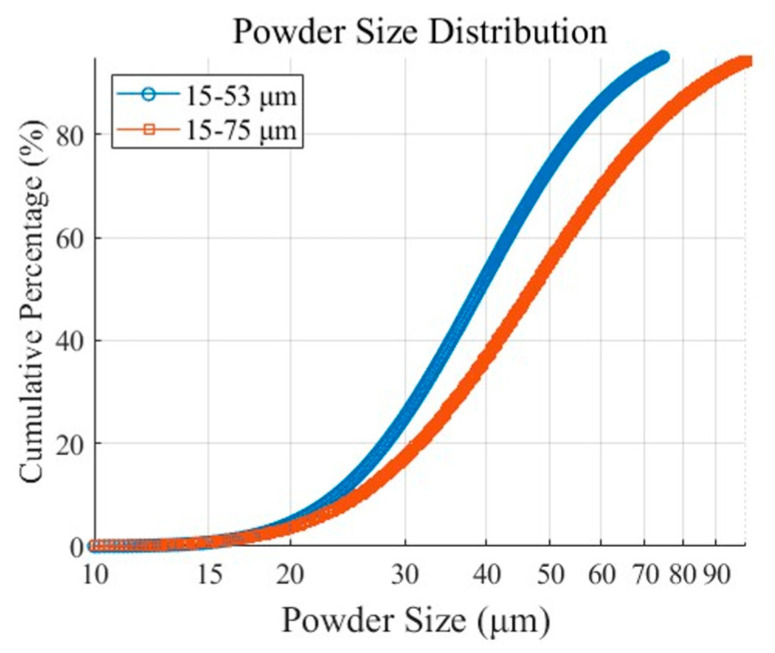
Cumulative powder size distribution of 15–53 μm and 15–75 μm powders.

The feasibility of utilizing the expanded PSD without compromising mechanical properties is a primary focus of this study. Experiments were conducted to evaluate the mechanical performance of the expanded PSD and its impact on cost efficiency and process stability. Figure 1 illustrates the powder size distribution differences between the two PSD ranges.

### 2.3. Parameter Optimization

New parameters for the expanded PSD were developed based on those from the original PSD. Since the chemical composition of Ti-6Al-4V remained unchanged, adjustments were made to account for changes in energy density caused by the expanded PSD instead of directly applying the existing parameters.

First, the layer thickness was kept at 60 µm, and laser power, scan speed, and trace spacing were adjusted to effectively melt the different powder sizes. Specifically, the expanded PSD includes larger powder sizes, requiring a higher energy density than the original PSD. To address this, the optimization process was conducted using a design of experiments (DoE) approach. This method enabled the systematic analysis of the influence of multiple parameters on the final part properties. By varying laser power, scan speed, and trace spacing within predefined ranges, the internal density of the samples was evaluated. The scan speed reduction of 100 mm/s was identified as optimal based on its ability to achieve a high internal density while maintaining melt pool stability and minimizing defects.

Contour parameter optimization involved adjusting both laser power and scan speed to enhance surface quality. Increased laser output played a key role in enhancing surface finish quality, as more energy was needed to melt the larger powders in the expanded PSD.

### 2.4. Mechanical Property Test

In this study, optimized process parameters for the expanded PSD were applied to Ti-6Al-4V grade 5 specimens, and their mechanical properties were evaluated and compared with those of specimens produced using the original PSD. Tension, compression, shear, and bearing tests were conducted to verify the mechanical properties, with all tests performed in accordance with ASTM standards.

All specimens were manufactured under identical process conditions, and the shape and arrangement of the specimens followed ASTM standards to ensure the reliability of the tests. The tests were conducted at a Korea Laboratory Accreditation Scheme (KOLAS) certified testing facility, and the experimental results were used to compare the mechanical properties of the expanded PSD with those of the original PSD.

**Figure 2:**
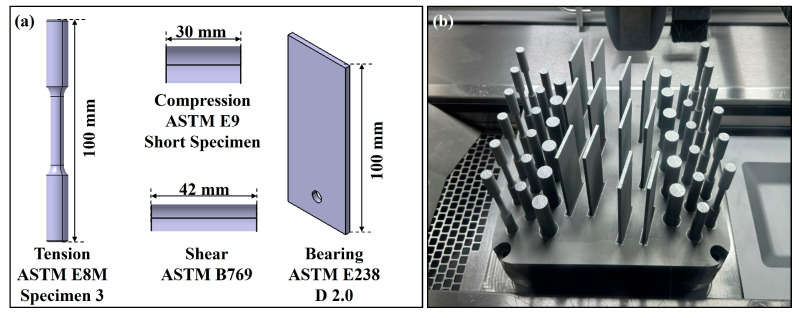
(**a**) Specimen design dimensions according to ASTM standards, (**b**) shape and arrangement of the manufactured specimens.

Figure 2 shows the shape and arrangement of the specimens used in the experiments.

### 2.5. Statistical Analysis for Reliability Assurance

To verify the stability and reliability of the mechanical properties observed in the expanded PSD specimens, statistical methods were applied. The Anderson–Darling test, a robust goodness-of-fit test, was used to evaluate the distribution of the mechanical property data and identify the most appropriate probability distribution for the dataset [36,37]. This test minimizes variability by selecting the distribution with the lowest test statistic as optimal, ensuring reliable analysis [38].

Equations (1)–(3) below represent the probability density function (PDF), cumulative distribution function (CDF), and survival function (SF) for a three-parameter Weibull distribution, where *α*, *β* and *γ* represent the scale, shape, and position parameters, respectively [39].

(1) $$f\left(x\right)=\left(\frac{\beta }{\alpha }\right)\cdot {\left(\frac{x-\gamma }{\alpha }\right)}^{\beta -1}\cdot e^{-{\left(\frac{x-\gamma }{\alpha }\right)}^{\beta }}$$

(2) $$F\left(x\right)=e^{-{\left(\frac{x-\gamma }{\alpha }\right)}^{\beta }}$$

(3) $$R\left(x\right)=1-e^{-{\left(\frac{t-\gamma }{\alpha }\right)}^{\beta }}$$

**Figure 3:**
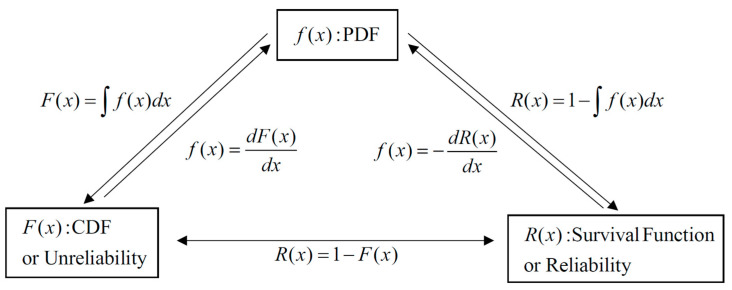
Relationship between the functions.

Figure 3 illustrates the relationship between the PDF, CDF, and SF. The PDF provides the likelihood of specific property values, while the CDF and SF indicate cumulative probabilities and the probability of exceeding a given threshold, respectively [40].

The SF was specifically used to assess compliance with the Metallic Materials Properties Development and Standardization (MMPDS) standards, as it is more intuitive than other methods, such as PDF or CDF. This calculation confirmed that the expanded PSD achieved consistent mechanical performance within MMPDS criteria [39,41].

## 3. Results

### 3.1. Parameter Optimization Results

Optimized parameters for the additive manufacturing of Ti-6Al-4V grade 5 material with the expanded PSD were derived through repeated experiments and density analysis. The final parameters are summarized in Table 3, and the differences were analyzed by comparing them with the original PSD.

The spot size mentioned in Table 3 refers to the diameter of the laser used for melting the material. Trace spacing represents the spacing between laser paths, laser power denotes the output of the laser, and scan speed indicates the speed of the laser’s movement along its path. However, these parameters were provided by the equipment manufacturer, and due to confidentiality, the exact values cannot be disclosed. Therefore, the values are represented using letters such as A, B, …, H, as described above. In the hatching parameter study, spot size, trace spacing, and laser power demonstrated the same values as the original parameters. but it was necessary to adjust the scan speed for the expanded PSD. Since the expanded PSD includes larger particle sizes, it requires a higher energy density compared to the original PSD. To address this, the scan speed was reduced by 100 mm/s. This adjustment was essential to ensure complete melting of the larger particles and to maintain the density of the part.

In the contour parameter optimization process, the laser power and scan speed were combined to adjust the energy density in order to improve surface quality. The increase in laser power contributed to melting the larger particles in the expanded PSD, leading to improved surface finish quality. Specifically, during this process, the optimal parameters were developed to improve both surface roughness and density simultaneously.

One of the main findings of this study is that additive manufacturing with the expanded PSD requires higher energy density compared to the original PSD. This parameter optimization plays a crucial role in maintaining process stability and quality when applying the expanded PSD. Table 3 shows the differences between the optimized parameters used for the original PSD and the expanded PSD.

### 3.2. Mechanical Property Test Results

Tension, compression, shear, and bearing tests were performed to evaluate the mechanical properties of Ti-6Al-4V grade 5 using the expanded PSD. The results were analyzed by comparing the mechanical properties of the original PSD and the expanded PSD. Although the expanded PSD showed slightly lower mechanical properties compared to the original PSD, all values exceeded MMPDS standards. The results of each test are summarized in Table 4.

#### 3.2.1. Tensile Test Result (ASTM E8)

**Figure 4:**
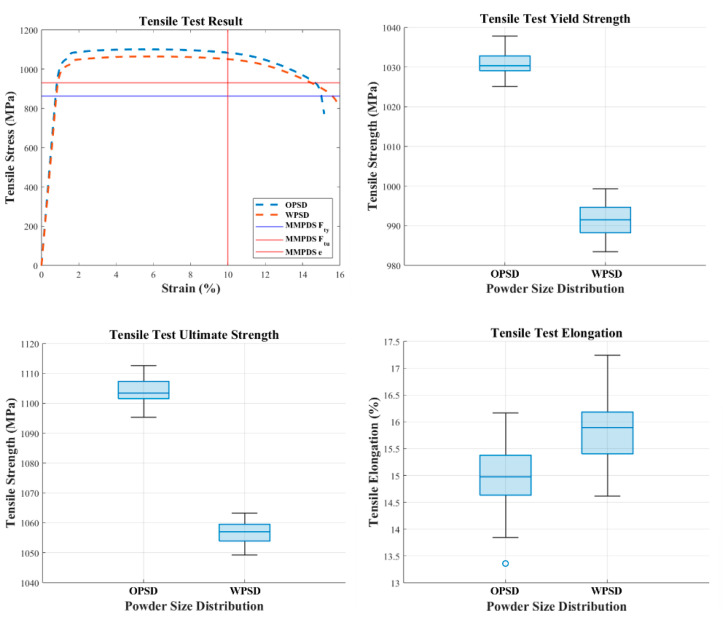
Comparison of tensile properties between OPSD and WPSD.

In the tensile test, the specimens made with the expanded PSD showed slightly lower yield strength and tensile ultimate strength compared to those made with the original PSD. The average yield strength of the expanded PSD specimens was 991 MPa, while their tensile ultimate strength was 1057 MPa. Compared to the original PSD specimens, which had an average yield strength of 1031 MPa and a tensile ultimate strength of 1104 MPa, the expanded PSD specimens showed a reduction of approximately 3.9% in yield strength and 4.3% in tensile ultimate strength, respectively. On the other hand, the elongation of the expanded PSD specimens slightly increased, indicating that the larger particles may induce more flexible deformation during the melting process. Figure 4 provides a visual comparison of the tensile properties between the original PSD and the expanded (wide) PSD specimens, where OPSD represents the results for the original PSD specimens and WPSD represents the results for the expanded (wide) PSD specimens.

#### 3.2.2. Compression Test Result (ASTM E9)

**Figure 5:**
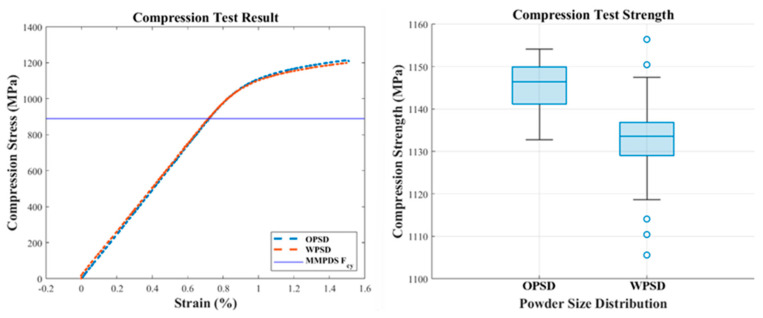
Comparison of compressive properties between OPSD and WPSD.

The compression test results showed that the expanded PSD specimens had slightly lower compressive yield strength than the original PSD specimens. The average compressive yield strength of the expanded PSD specimens was measured at 1132 MPa, approximately 1.1% lower than the 1145 MPa recorded for the original PSD specimens. This indicates that the bonding strength of the larger particles in the expanded PSD is slightly lower than that of the original PSD. Figure 5 visually compares the compressive properties between the original PSD and the expanded (wide) PSD specimens.

#### 3.2.3. Shear Test Result (ASTM B769)

**Figure 6:**
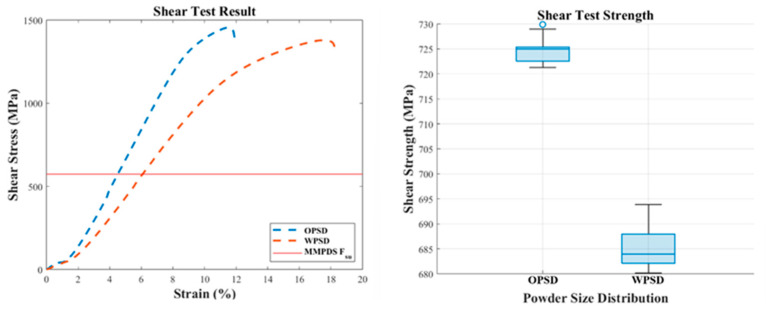
Comparison of shear properties between OPSD and WPSD.

The shear test results showed that the specimens made with the expanded PSD had slightly lower shear strength compared to the original PSD specimens. The average shear strength of the expanded PSD specimens was 685 MPa, which is around 5.5% lower than the 725 MPa measured for the original PSD specimens. This indicates that the expanded PSD exhibits slightly lower stiffness under shear loads compared to the original PSD. Figure 6 presents a visual comparison of the shear properties for the original and expanded PSD specimens.

#### 3.2.4. Pin-Type Bearing Test Result (ASTM E238)

**Figure 7:**
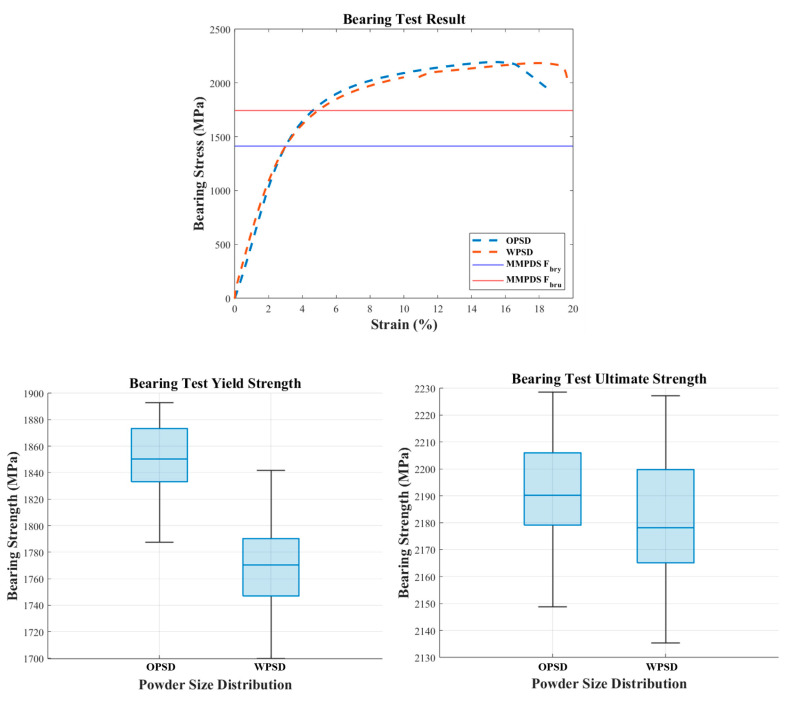
Comparison of bearing properties between OPSD and WPSD.

The bearing test results showed that the bearing yield strength of the expanded PSD specimens was slightly lower compared to the original PSD specimens. The average yield bearing strength of the expanded PSD specimens was 1767 MPa, approximately 4.5% lower than the 1850 MPa recorded for the original PSD specimens. The maximum bearing strength showed a similar trend. Figure 7 illustrates the bearing properties, allowing a direct comparison between the original PSD and expanded PSD specimens.

### 3.3. Statistical Analysis for Reliability Assurance Result

The statistical methods described in Section 2.5 were applied to assess the reliability of the mechanical properties of the expanded PSD specimens. The Anderson–Darling test indicated that the experimental data fit well with the 3-parameter Weibull distribution.

Among the analysis methods available, including PDF, CDF, and SF, the SF was selected as the primary tool for presenting the results. This decision was based on the need to simplify the presentation and avoid redundancy, as PDF, CDF, and SF are interrelated and yield overlapping insights. The use of SF provided a clear and direct visualization of the probability trends, particularly in demonstrating that the probability of mechanical properties falling below the MMPDS standards was negligible.

The following analysis results offer a detailed comparison of the property distributions and stability between the original and expanded PSDs.

#### 3.3.1. Statistically Analyzed Tensile Test Results

**Figure 8:**
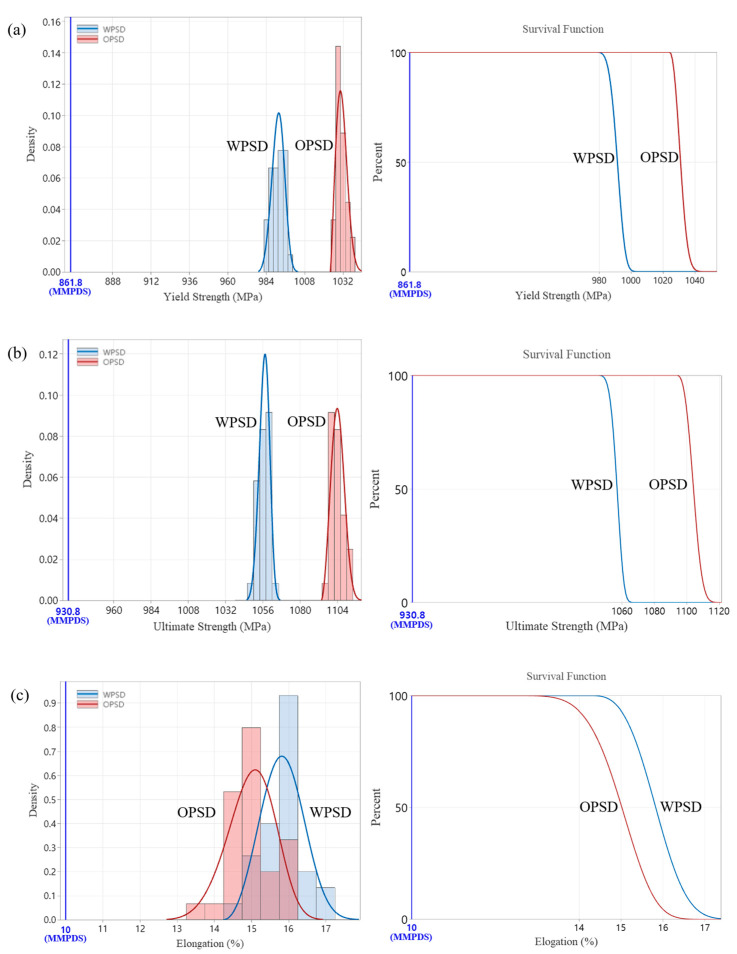
Statistically analyzed tensile test results, (**a**) yield strength, (**b**) ultimate strength, (**c**) elongation.

The Anderson–Darling test was used to estimate the data distribution shape for each test result, and this distribution was fitted to the property data. The results showed statistically significant differences in yield strength and tensile ultimate strength between the original PSD and the expanded PSD. As shown in Figure 8, the expanded PSD exhibited a survival curve similar to that of the original PSD, with both distributions demonstrating compliance with the MMPDS standard with a probability close to 100%. In particular, the elongation data showed similar distribution patterns between the two PSDs.

#### 3.3.2. Statistically Analyzed Compression Test Results

**Figure 9:**
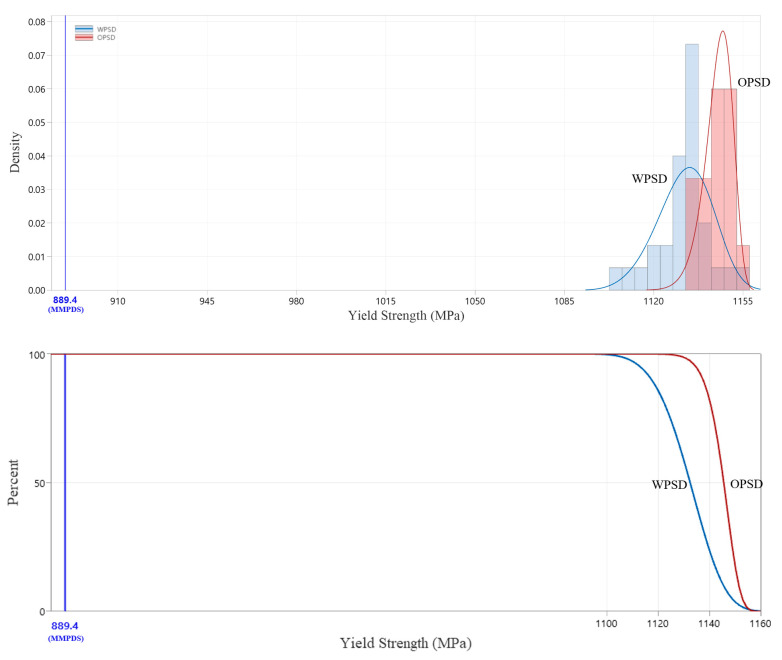
Statistically analyzed compression test results.

Compression test results, analyzed with a 3-parameter Weibull distribution, revealed slight differences in compressive yield strength between the original and expanded PSDs. As shown in Figure 9, the expanded PSD maintained compression characteristics like those of the original PSD, with both distributions demonstrating significant compliance with the MMPDS standard. The survival function analysis indicated a very low probability of falling below the MMPDS standard threshold.

#### 3.3.3. Statistically Analyzed Shear Test Results

**Figure 10:**
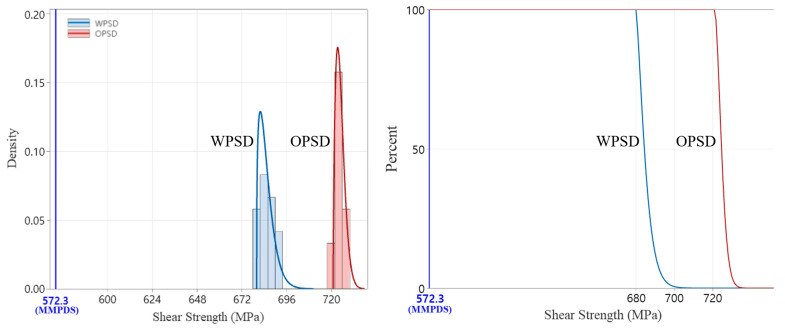
Statistically analyzed shear test results.

Shear test results revealed a significant difference between the original PSD and the expanded PSD. As seen in Figure 10, the expanded PSD exhibited slightly lower shear strength compared to the original PSD. However, the survival function analysis confirmed compliance of the expanded PSD with the MMPDS standard. Both powders maintained shear strength characteristics that met and exceeded the MMPDS requirements.

#### 3.3.4. Statistically Analyzed Bearing Test Results

**Figure 11:**
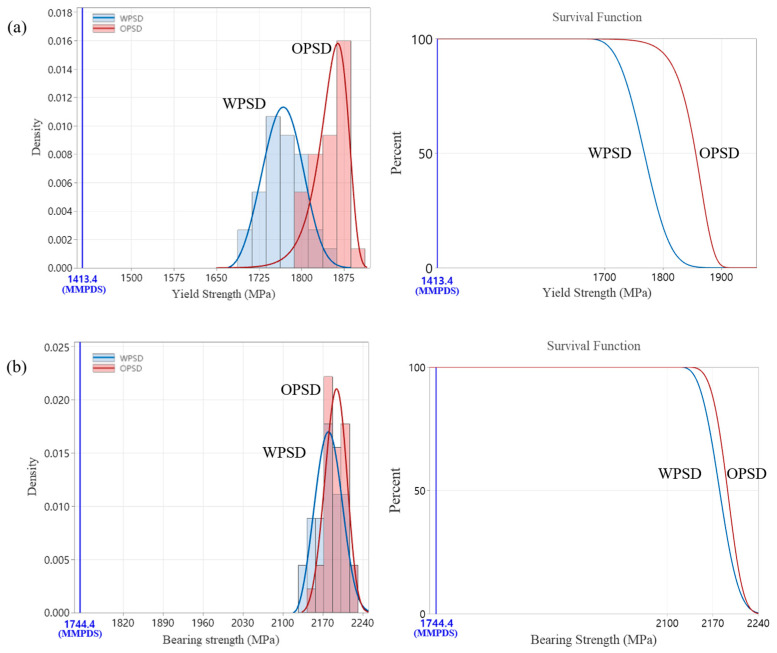
Statistically analyzed bearing test results, (**a**) yield strength, (**b**) ultimate strength.

Bearing test results revealed some differences between the original and expanded PSDs, but survival function analysis confirmed that both PSDs comply with the MMPDS standard. As seen in Figure 11, the expanded PSD maintained similar bearing yield strength and maximum bearing strength to the original PSD, with both powders demonstrating reliable mechanical performance.

## 4. Discussion

This study investigated the feasibility of expanding the PSD of Ti-6Al-4V grade 5 from 15–53 μm to 15–75 μm to improve cost efficiency and process flexibility in L-PBF. The experimental results demonstrated that the mechanical properties of specimens produced using the expanded PSD met MMPDS standards, despite minor reductions in strength. Specifically, the tensile yield strength decreased by 3.9%, compressive strength by 1.1%, shear strength by 5.5%, and bearing yield strength by 4.5%. These reductions, though minimal, underline the necessity of understanding the underlying mechanisms associated with PSD expansion.

The decrease in strength can be attributed to several factors. Firstly, the inclusion of larger particles in the PSD appears to influence microstructural characteristics. Larger particles promote the formation of coarser grains during solidification, which typically reduces material strength. Additionally, the relaxation of residual stresses within the material, caused by slower cooling rates associated with larger melt pools, may have contributed to the observed mechanical performance. These findings align with prior studies [42], which highlighted the correlation between grain size and mechanical properties in titanium alloys.

A slight increase in oxygen content, observed in the expanded PSD powders, also likely influenced material properties. Oxygen, a known interstitial element in titanium alloys, can enhance strength but reduce ductility [43]. The marginally higher oxygen levels in the expanded PSD specimens could have reinforced this trade-off, where ductility improved slightly but strength decreased. This emphasizes the need for strict control over powder handling and storage conditions, particularly during recycling or reuse, to mitigate oxygen uptake.

Statistical analysis using the Anderson–Darling test confirmed that the variability in mechanical properties between the original and expanded PSD specimens was minimal, indicating consistent performance.

Lastly, the broader PSD reduced material waste by minimizing the need for sieving and filtration, directly contributing to increased production yield. This reduction in waste aligns with sustainable manufacturing objectives, as less powder is discarded during production. However, the slight trade-off in mechanical performance highlights the importance of parameter optimization to fully harness the potential benefits of expanded PSDs while maintaining or even improving mechanical properties.

## 5. Conclusions

This study demonstrated that expanding the PSD of Ti-6Al-4V grade 5 from 15–53 μm to 15–75 μm offers a viable approach to reducing costs and improving process flexibility in L-PBF applications. Despite minor reductions in mechanical strength, all tested properties complied with MMPDS standards, indicating that the expanded PSD can meet the rigorous requirements of high-performance industries.

The observed reductions in strength, coupled with a slight increase in elongation, can be attributed to factors such as larger particle sizes, grain growth, residual stress relaxation, and marginally lower oxygen content. While the findings highlight the potential advantages of cost reduction and process flexibility, the potential drawbacks of PSD expansion, such as its impact on fatigue life, wear resistance, and other critical properties, require further investigation.

Future research should validate these results through large-scale production trials and include a detailed microstructural analysis of the mechanical property variations observed in the tested specimens. Such studies should also address the broader implications of PSD expansion, including both its benefits and potential limitations, to ensure a balanced understanding of its suitability for high-performance applications.

## Figures and Tables

**Table 1:** GE additive M2 machine specifications.

Parameter	Value
Maximum laser power (P)	400 W
Maximum scan speed (V)	4500 mm/s
Focus diameter (D)	70–500 µm
Layer thickness (t)	25–100 µm

**Table 2:** Ti-6Al-4V grade 5 powder material chemical composition.

Item	Unit	Minimum Limit	Maximum Limit	Testing Method	Status
Powder composition					
Al	Wt. %	5.50	6.75	ASTM E2371-21 [33]	Conforming
V	Wt. %	3.50	4.50	ASTM E2371-21	Conforming
Fe	Wt. %	-	0.30	ASTM E2371-21	Conforming
O	Wt. %	-	0.20	ASTM E1409-13 [34]	Conforming
C	Wt. %	-	0.08	ASTM E1941-10 [35]	Conforming
N	Wt. %	-	0.05	ASTM E1409-13	Conforming
H	Wt. %	-	0.015	ASTM E1447-09	Conforming
Y	Wt. %	-	0.005	ASTM E2371-21	Conforming
Other Each	Wt. %	-	0.10	ASTM E2371-21	Conforming
Other Total	Wt. %	-	0.40	ASTM E2371-21	Conforming
Ti	Wt. %	Balance	Balance	-	-

**Table 3:** Parameter for optimized wide PSD powders.

	Original PSD (15–53 μm)	Expanded PSD (15–75 μm)
Layer thickness (µm)	60	60
Hatching parameter		
Spot size (µm)	A	A
Trace spacing (µm)	B	B
Laser power (W)	C	C
Scan speed (mm/s)	D	D − 100
Contour parameter		
Spot size (µm)	E	E
Trace spacing (µm)	F	F
Laser power (W)	G	G + 20
Scan speed (mm/s)	H	H

**Table 4:** The average mechanical properties of 30 specimens for both the original PSD and expanded PSD powders, using the MMPDS standards as a benchmark.

	MMPDS	Original PSD (15–53 μm)	Expanded PSD (15–75 μm)
Tensile yield strength (*F_ty_*, MPa)	861.9	1031	991.3
Tensile ultimate strength (*F_tu_*, MPa)	930.8	1104	1057
Tensile elongation (e, %)	10.00	14.99	15.84
Compressive yield strength (*F_cy_*, MPa)	889.4	1145	1132
Shear strength (*F_su_*, MPa)	572.3	724.9	685.3
Bearing yield strength (*F_bry_*, MPa)	1413	1850	1767
Bearing ultimate strength (*F_bru_*, MPa)	1744	2192	2182

## Data Availability

The original contributions presented in this study are included in the article. Manufacturing parameters are not available due to institutional security restrictions. Specimen test data are stored on the institution’s NAS and are not publicly accessible; however, they can be shared with individuals upon reasonable request directed to the corresponding author.
